# Impact of frailty on mortality and quality of life in patients with a history of cancer undergoing transcatheter aortic valve replacement

**DOI:** 10.1002/clc.23927

**Published:** 2022-10-04

**Authors:** Nikitha Kosaraju, Perry Wu, Mei Leng, Marielle Bolano, Asim M. Rafique, John Shen, Nancy Satou, Jeanne Huchting, Deena Goldwater, Olcay Aksoy, Eric H. Yang

**Affiliations:** ^1^ David Geffen School of Medicine University of California at Los Angeles Los Angeles California USA; ^2^ Department of Medicine, Division of Cardiology University of California at Irvine Orange California USA; ^3^ Department of Medicine, Division of General Internal Medicine and Health Services Research University of California at Los Angeles Los Angeles California USA; ^4^ Department of Medicine, Division of Geriatrics University of California at Los Angeles Los Angeles California USA; ^5^ Department of Medicine, Division of Cardiology University of California at Los Angeles Los Angeles California USA; ^6^ Department of Medicine, Division of Hematology/Oncology University of California at Los Angeles Los Angeles California USA; ^7^ Department of Surgery, Division of Cardiac Surgery University of California at Los Angeles Los Angeles California USA; ^8^ Welcome Health Medical Group Welcome Health Inc Long Beach California USA; ^9^ UCLA Cardio‐Oncology Program, Department of Medicine, Division of Cardiology University of California at Los Angeles Los Angeles California USA

**Keywords:** cardio‐oncology, KCCQ, readmission, structural heart disease, transcatheter aortic valve replacement

## Abstract

**Background:**

Transcatheter aortic valve replacement (TAVR) is increasingly offered for aortic stenosis (AS) treatment in patients with a history of cancer. The impact of frailty on outcomes in this specific patient population is not well described.

**Hypothesis:**

Frailty is associated with mortality and poorer quality of life (QOL) outcomes in patients undergoing TAVR with a history of cancer.

**Methods:**

This retrospective single center cohort study included AS patients who underwent TAVR from August 1, 2012 to May 15, 2020. Frailty was measured using serum albumin, hemoglobin, gait speed, functional dependence, and cognitive impairment. The primary outcome was a composite of all‐cause mortality and QOL at 1 year. A poor primary outcome was defined as either all‐cause mortality, Kansas City Cardiomyopathy Questionnaire overall summary (KCCQ‐OS) score <45 or a KCCQ‐OS score decline of ≥10 points from baseline. Regression analysis was used to determine the impact of frailty on the primary outcome.

**Results:**

The study population was stratified into active/recent cancer (*n* = 107), remote cancer (*n* = 85), and non‐cancer (*n* = 448). Univariate analysis of each cohort showed that frailty was associated with the primary outcome only in the non‐cancer cohort (*p* = .004). Multivariate analysis showed that cancer history was not associated with a poor primary outcome, whereas frailty was (1.7 odds ratio, 95% confidence interval [CI]: 1.1–2.8; *p* = .028).

**Conclusions:**

Frailty is associated with mortality and poor QOL in the overall and non‐cancer cohorts. Further investigation is warranted to understand frailty's effect on the cancer population. Frailty should be heavily considered during TAVR evaluation.

**Abbreviations**
ADL: activities of daily living
AS: aortic stenosis
EFT: essential frailty toolset
KCCQ: Kansas City Cardiomyopathy Questionnaire
NYHA: New York Heart Association
QOL: quality of life
STS: Society of Thoracic Surgeons
TAVR: transcatheter aortic valve replacement
TVT: transcatheter valve therapy

## 1 INTRODUCTION

Aortic stenosis (AS) disproportionately affects older adults in developed countries, a phenomenon driven primarily by an increased prevalence of degenerative calcific aortic valve stenosis with age.^1^, ^2^ Other common predisposing factors for cancer and cardiovascular disease such as diet, exercise, body mass index, and smoking have also contributed to an estimated prevalence of cancer in severe AS patients ranging from 19.2% to 26.6%.^3^, ^4^ Pivotal randomized controlled trials show transcatheter aortic valve replacement (TAVR) to be either non‐inferior or superior to surgical aortic valve replacement for patients with symptomatic severe AS across the spectrum of surgical risk.^5^, ^6^, ^7^, ^8^, ^9^, ^10^ TAVR may also be preferable in patients with active cancers and unclear prognoses, to mitigate cardiovascular complications while undergoing cancer‐directed treatment.^11^ A recent meta‐analysis of outcomes in patients with a history of cancer undergoing TAVR showed that a cancer history was not associated with poorer short‐ or long‐term survival.^12^ However, these previous studies did not look at frailty, a known major determinant of outcomes post‐TAVR.^13^, ^14^, ^15^ Since TAVR patients are often older and have multiple comorbidities, it is important to consider both survival and quality of life (QOL) when assessing outcomes.^13^, ^16^, ^17^ Given that frailty is known to impact outcomes in treatments related to cardiology and to oncology patients, we sought to assess the impact of frailty on a composite outcome of mortality and QOL and on readmission rates in the AS population with a history of cancer undergoing TAVR.^18^, ^19^ We hypothesized that with significant metrics of frailty in patients with a history of active or remote cancer, frailty would be associated with increased mortality, decreased QOL, and increased readmission rates in this population.

## 2 METHODS

### 2.1 Study design, sample, and data

This was a single center retrospective cohort study comparing outcomes of patients with (1) active cancer to those of patients with a (2) remote history of cancer to those of patients with (3) no history of cancer who underwent TAVR for AS. All patients were cared for at the University of California, Los Angeles (UCLA) Ronald Reagan Medical Center between August 1, 2012 to May 15, 2020. This study was approved by the Institutional Review Board (IRB#16‐000680). UCLA is an academic quaternary care hospital with a National Cancer Institute‐designated cancer center. Study subjects were included in the ACC Transcatheter Valve Therapy (TVT) registry which includes several key patient characteristics, procedure details, and post procedural outcomes. Patients with a predicted overall survival of <12 months or for whom minimal improvement in QOL was anticipated were disqualified from TAVR as per 2020 ACC/AHA guidelines. Eight hundred and thirty‐seven consecutive patients were identified from the study period. Inclusion criteria included undergoing a TAVR for severe AS. Exclusion criteria included unsuccessful device implantation (i.e., aborted or converted to open heart surgery), valve‐in‐valve procedures, incomplete follow‐up, or unverifiable cancer status. The final cohort for analysis consisted of 640 patients (Supporting Information: Figure 1).

Patient demographics, baseline metrics and health characteristics, Kansas City Cardiomyopathy Questionnaire (KCCQ) scores, frailty metrics, readmission data, and outcome data were extracted from the TVT registry. STS scores from the TVT registry were designated into three categories: (1) high‐risk (STS ≥ 8%), (2) intermediate‐risk (STS ≥ 4% and <8%), and (3) low‐risk (STS < 4%) to facilitate regression analysis. Frailty metrics obtained from the TVT registry included serum albumin, hemoglobin, and gait speed. Per the TVT registry, readmissions were categorized as either being non‐valve‐related readmissions or valve‐related readmissions. Cancer history, treatment history, and additional frailty metrics such as cognitive impairment and functional dependence were obtained through chart review of the electronic health record (EHR). Cancer staging that was not explicitly documented in the EHR by an oncologist was verified using two‐physician review. Patient data were entered into a deidentified Research Electronic Data Capture (REDCap, Vanderbilt University, Nashville, TN, USA) database hosted at UCLA.^20^, ^21^

### 2.2 Cancer stratification, frailty assessment, and readmission rates

Patients were categorized into three cohorts: (1) active or recent history of cancer (achieved remission <5 years ago), (2) remote history of cancer (achieved remission ≥5 years ago), and (3) no history of cancer. A composite frailty score (CFS) was created using serum albumin, hemoglobin, gait speed, cognitive impairment, and functional dependence. These five markers were chosen as a modified Essential Frailty Toolset (EFT), with an addition of functional dependence and gait speed used in lieu of chair rise.^14^, ^22^ The CFS was derived accordingly: (1) lowest quartiles of gait speed, serum albumin, and hemoglobin were each assigned a value of 1; (2) presence of cognitive impairment and functional dependence were each assigned a value of 1. A CFS of 0 was considered the least frail and a 5 was considered the most frail. Patients were dichotomized to high frailty (CFS 3–5) and low frailty (CFS 0–2) to facilitate multivariate analysis. Readmission rates were defined as the percent of patients who were readmitted between procedure date and either their 30‐day or 1‐year follow‐up date. Additional details can be found in the Supplement.

### 2.3 Evaluation of outcomes

The KCCQ is a patient‐reported, disease‐specific health status survey and the primary health status instrument for the TVT registry.^17^, ^23^ The KCCQ overall summary (KCCQ‐OS) score ranges from 0 to 100 with higher scores translating to less symptom burden and better QOL.^17^ A KCCQ‐OS score <45 correlates to a New York Heart Association (NYHA) functional classification of III/IV.^24^, ^25^, ^26^

The primary outcome of this study was defined as a composite outcome of both all‐cause mortality and QOL at 1 year. A poor QOL outcome was defined as a KCCQ‐OS score <45 at the time of follow‐up, or a decrease in KCCQ‐OS score by ≥10 points from baseline.^13^, ^23^, ^27^ Secondary outcomes included (1) the composite outcome at 30 days, (2) all‐cause mortality at 30 days and 1 year, (3) QOL at 30 days and 1 year, and (4) TVT‐reported all‐cause readmission rates at 30 days and 1 year. Of note, the 30‐day and 1‐ year follow‐up timepoints coincided with mandated follow‐up as per TVT registry requirements.

### 2.4 Statistical analysis

Baseline characteristics were presented as mean ± SD for continuous variables and as frequency (percentage) for categorical variables. ANOVA test was used to compare continuous variables and the *χ* ^2^ test was used to compare categorical variables. Univariate logistic regressions stratified by cancer status were done with the CFS and individual frailty markers, followed by multivariate logistic regressions to show relations. All tests were two‐sided and *p* < .05 was considered statistically significant. All analyses were performed with SAS v9.4 (The SAS Institute).

## 3 RESULTS

### 3.1 Baseline characteristics

In this study of 640 patients, 107 had active/recent cancer, 85 had remote cancer, and 448 had no history of cancer. Baseline demographic and clinical characteristics stratified by cancer status are presented in Table 1 and Supporting Information: Table 1. The remote cancer patients were significantly older than the active/recent cancer patients and the non‐cancer patients (82.7 ± 8.9 vs. 80.8 ± 9.7 and 79.7 ± 10.9%, respectively, *p* = .043). Of note, diabetes and a history of atrial fibrillation/flutter were also significantly different between the three cohorts (*p* < .050). As expected, active/recent cancer patients were more immunocompromised—defined as taking immunosuppressive medication therapy such as systemic steroid therapy, anti‐rejection medications, and/or chemotherapy—than remote cancer and non‐cancer patients (16.8% vs. 4.7% and 5.1%, respectively, *p* < .001). There were no significant differences between the three cohorts when comparing other baseline characteristics including STS scores, hypertension, NYHA functional class, or KCCQ‐OS scores. Specific data regarding baseline malignancy characteristics of the active/recent cancer and remote cancer cohorts are summarized in Table 2.

**Table 1:** Baseline patient characteristics stratified by cancer status

	Active/Recent cancer (*n* = 107)	Remote cancer (*n* = 85)	No cancer (*n* = 448)	*p* value
Age, years (mean)	80.8 ± 9.7	82.7 ± 8.9	79.7 ± 10.9	0.043*
Female	42 (39.3%)	42 (49.4%)	204 (45.5%)	0.342
White race	98 (91.6%)	77 (90.6%)	387 (86.4%)	0.236
Weight, kg (mean)	72.8 ± 19.4	74.5 ± 19.7	75.2 ± 19.3	0.522
Hypertension	94 (87.9%)	73 (85.9%)	400 (89.3%)	0.641
Dyslipidemia	101 (94.4%)	81 (95.3%)	419 (93.5%)	0.801
Prior myocardial infarction	24 (22.9%)	17 (20.2%)	118 (26.8%)	0.374
Prior CABG surgery	15 (14.0%)	7 (8.2%)	73 (16.3%)	0.152
Prior peripheral arterial disease	21 (19.6%)	23 (27.1%)	129 (28.9%)	0.155
Prior stroke	10 (9.4%)	8 (9.4%)	43 (9.6%)	0.996
Atrial fibrillation/flutter	50 (46.7%)	42 (50%)	152 (34.0%)	0.003*
Permanent pacemaker	12 (11.2%)	14 (16.5%)	53 (11.8%)	0.455
Diabetes mellitus	19 (17.8%)	17 (20%)	164 (36.6%)	<0.001*
Left ventricular ejection fraction, %	55.2 ± 14.6	54.9 ± 14.2	54.9 ± 15.3	0.987
NYHA functional class				0.888
Class I	12 (11.3%)	7 (8.2%)	39 (8.8%)	
Class II	50 (47.2%)	39 (45.9%)	207 (46.7%)	
Class III	39 (36.8%)	35 (41.2%)	165 (37.3%)	
Class IV	5 (4.7%)	4 (4.7%)	32 (7.2%)	
Chronic lung disease				0.255
None	73 (68.2%)	52 (63.4%)	318 (71.5%)	
Mild	18 (16.8%)	22 (26.8%)	67 (15.1%)	
Moderate	10 (9.4%)	5 (6.1%)	33 (7.4%)	
Severe	6 (5.6%)	3 (3.7%)	27 (6.1%)	
Home oxygen	6 (5.6%)	4 (4.7%)	27 (6.0%)	0.889
Current/recent smoker (within 1 year)	4 (3.7%)	2 (2.4%)	11 (2.5%)	0.688
Platelets, microliters (mean)	215,019 ± 109,067	191,333 ± 74,864	196,166 ± 80,194	0.087
Creatinine, mg/dl (mean)	1.2 ± 0.9	1.3 ± 0.9	1.4 ± 1.4	0.281
Current dialysis	3 (2.8%)	4 (4.7%)	25 (5.6%)	0.492
Immunocompromised	18 (16.82%)	4 (4.71%)	23 (5.13%)	<0.001*
STS risk score, % (mean)	6.5 ± 5.0	6.5 ± 4.8	6.4 ± 4.1	0.966
STS risk score, categorical				0.637
Low risk (STS < 4%)	38 (36.9%)	27 (33.3%)	125 (29.3%)	
Intermediate risk (STS ≥ 4% and <8%)	38 (36.9%)	31 (38.3%)	180 (42.3%)	
High risk (STS ≥ 8%)	27 (26.2%)	23 (28.4%)	121 (28.4%)	
Baseline KCCQ score (mean)	48.1 ± 25.3	45.2 ± 24.9	48.7 ± 25.4	0.511

*Denotes statistically significant *p* values (*p* ≤ .05).

Abbreviations: CABG, coronary artery bypass graft; KCCQ, Kansas City Cardiomyopathy Questionnaire; NYHA, New York Heart Association; STS, Society of Thoracic Surgeons.

**Table 2:** Baseline cancer characteristics of the active/recent cancer and remote cancer cohorts

	Active/Recent cancer (*n* = 107)	Remote cancer (*n* = 85)	*p* value
Cancer type			
Multiple cancers	29 (27.1%)	4 (4.7%)	0.147
Prostate cancer	22 (20.6%)	14 (16.5%)	0.471
Breast cancer	19 (17.8%)	24 (28.2%)	0.084
Melanoma	12 (11.2%)	6 (7.1%)	0.326
Bladder cancer	10 (9.3%)	4 (4.7%)	0.219
Lung cancer	8 (7.5%)	2 (2.4%)	0.19
Myelodysplastic syndromes	8 (7.5%)	0 (0%)	0.009*
Chronic lymphocytic leukemia	8 (7.5%)	0 (0%)	0.009*
Colon cancer	7 (6.5%)	10 (11.8%)	0.206
Non‐Hodgkin lymphoma	7 (6.5%)	3 (3.5%)	0.517
Hodgkin lymphoma	5 (4.7%)	5 (5.9%)	0.752
Liver cancer	5 (4.7%)	0 (0%)	0.067
Renal cancer	4 (3.7%)	4 (4.7%)	0.734
Rectal cancer	2 (1.9%)	3 (3.5%)	0.657
Ovarian/fallopian cancer	2 (1.9%)	3 (3.5%)	0.657
Gastric cancer	2 (1.9%)	2 (2.4%)	1
Gastrointestinal lymphoma	2 (1.9%)	1 (1.2%)	1
Thyroid cancer	2 (1.9%)	0 (0%)	0.504
Essential thrombocytosis	2 (1.9%)	0 (0%)	0.504
Endometrial/uterine cancer	1 (0.9%)	2 (2.4%)	0.585
Throat cancer	1 (0.9%)	0 (0%)	1
Esophageal cancer	1 (0.9%)	0 (0%)	1
Parotid gland cancer	1 (0.9%)	0 (0%)	1
Multiple myeloma	1 (0.9%)	0 (0%)	1
Chronic myeloid leukemia	1 (0.9%)	0 (0%)	1
Acute promyelocytic leukemia	1 (0.9%)	0 (0%)	1
Other leukemia	1 (0.9%)	0 (0%)	1
Pancreatic cancer	1 (0.9%)	0 (0%)	1
Waldenstroms macroglobinemia	1 (0.9%)	0 (0%)	1
Polycythemia vera	1 (0.9%)	0 (0%)	1
Cervical cancer	0 (0%)	2 (2.4%)	0.195
Gallbladder cancer	0 (0%)	1 (1.2%)	0.443
Sinus cancer	0 (0%)	1 (1.2%)	0.443
Acute myeloid leukemia	0 (0%)	0 (0%)	N/A
Nonmelanoma skin cancer	0 (0%)	0 (0%)	N/A
Other	6 (5.6%)	1 (1.2%)	0.135
Solid versus liquid cancer			
Solid cancer	73 (68.2%)	74 (87.1%)	0.003*
Liquid cancer	23 (21.5%)	9 (10.6%)	
Multiple cancers	11 (10.3%)	1 (1.2%)	
Unknown	0 (0%)	1 (1.2%)	
Cancer stage			
Stage 0	6 (5.6%)	3 (3.5%)	<0.001*
Stage I	14 (13.1%)	16 (18.8%)	
Stage II	10 (9.3%)	6 (7.1%)	
Stage III	10 (9.3%)	4 (4.7%)	
Stage IV	20 (18.7%)	0 (0%)	
Presumed early (I–III cured)	9 (8.4%)	41 (48.2%)	
Presumed late (III/IV)	1 (0.9%)	0 (0%)	
Unknown	37 (34.6%)	15 (17.6%)	
Early (0, I, II, or presumed early)	39 (36.4%)	66 (77.6%)	<0.001*
Late (III, IV, or presumed late)	31 (29.0%)	4 (4.7%)	
Unknown	37 (34.6%)	15 (17.6%)	
Treatments			
Chest radiation	16 (15.0%)	22 (25.9%)	0.018*
Unknown	1 (0.9%)	11 (12.9%)	
Antineoplastic therapy	54 (50.5%)	23 (27.1%)	0.025*
Unknown	0 (0%)	16 (18.8%)	
Surgical excision	53 (49.5%)	64 (75.3%)	0.003*
Unknown	19 (17.8%)	6 (7.1%)	
Treatment goal			
Palliative	37 (34.6%)	1 (1.2%)	<0.001*
Curative	39 (36.4%)	81 (95.3%)	
Patient declined full treatment	2 (1.9%)	1 (1.2%)	
Undecided	22 (20.6%)	0 (0%)	
Unknown	7 (6.5%)	2 (2.4%)	

*Denotes statistically significant *p* values (*p* ≤ .05).

### 3.2 Outcomes

One‐year outcome data are depicted in Table 3a and Table 3b and the Central Illustration 1. The all‐cause mortality was 11.4% in the active/recent cancer cohort, 10% in the remote cancer cohort, and 9.5% in the non‐cancer cohort (*p* = .829). The percent of patients with a KCCQ‐OS score <45 or with a ≥10 point drop in KCCQ‐OS score from baseline was 12.5% in the active/recent cancer cohort, 8.5% in the remote cancer cohort, and 9.6% in the non‐cancer cohort (*p* = .739). Accordingly, there was no significant difference in the primary composite outcome between the three cohorts. However, readmission rates were significantly lower in the non‐cancer cohort when compared to the active/recent and remote cancer groups (25.2% vs. 36.5% and 35.3%, respectively, *p* = .022). Most of the readmissions in all cohorts were unrelated to TAVR complications (Supporting Information: Table 3).

**Table 3a:** One‐year outcomes stratified by cancer status

	Total population (*n* = 640)	Active/Recent cancer (*n* = 107)	Remote cancer (*n* = 85)	No cancer (*n* = 448)	*p* value
Mortality	61 (9.9%)	12 (11.4%)	8 (10%)	41 (9.5%)	0.829
KCCQ‐OS Score (mean)	83.0 ± 20.7	81.2 ± 20.4	79.3 ± 22.1	84.1 ± 20.5	0.258
Patients with KCCQ‐OS < 45	26 (7.2%)	6 (9.4%)	3 (6.4%)	17 (6.8%)	0.700
Patients with KCCQ‐OS Drop > 10	21 (6.1%)	5 (8.6%)	3 (6.5%)	13 (5.5%)	0.608
Readmission rate	182 (28.4%)	39 (36.5%)	30 (35.3%)	113 (25.2%)	0.022*
Patients with poor quality of life	36 (10.0%)	8 (12.5%)	4 (8.5%)	24 (9.6%)	0.739
Patients with poor primary composite outcomes	97 (15.7%)	20 (19.1%)	12 (14.8%)	65 (15.0%)	0.574

*Denotes statistically significant *p* values (*p* ≤ .05).

Abbreviation: KCCQ‐OS, Kansas City Cardiomyopathy Questionnaire overall summary.

**Table 3b:** One‐year outcomes comparing patients with cancer history to non‐cancer patients

	Active/Recent cancer versus No cancer	Remote cancer versus No cancer
	OR (95% CI) *p* value	OR (95% CI) *p* value
Mortality	1.2 (0.6–2.5) *p* = .623	1.1 (0.5–2.4) *p* = .916
Poor QOL	1.4 (0.6–3.2) *p* = .442	0.9 (0.3–2.7) *p* = .620
Composite outcome (mortality or poor QOL)	1.3 (0.8–2.3) *p* = .326	1.0 (0.5–1.9) *p* = .646
Readmission rate	1.7 (1.1–2.7) *p* = .020*	1.6 (1.0–2.7) *p* = .056

*Denotes statistically significant *p* values (*p* ≤ .05).

Abbreviations: CI, confidence interval; OR, odds ratio; QOL, quality of life.

**Figure 1:**
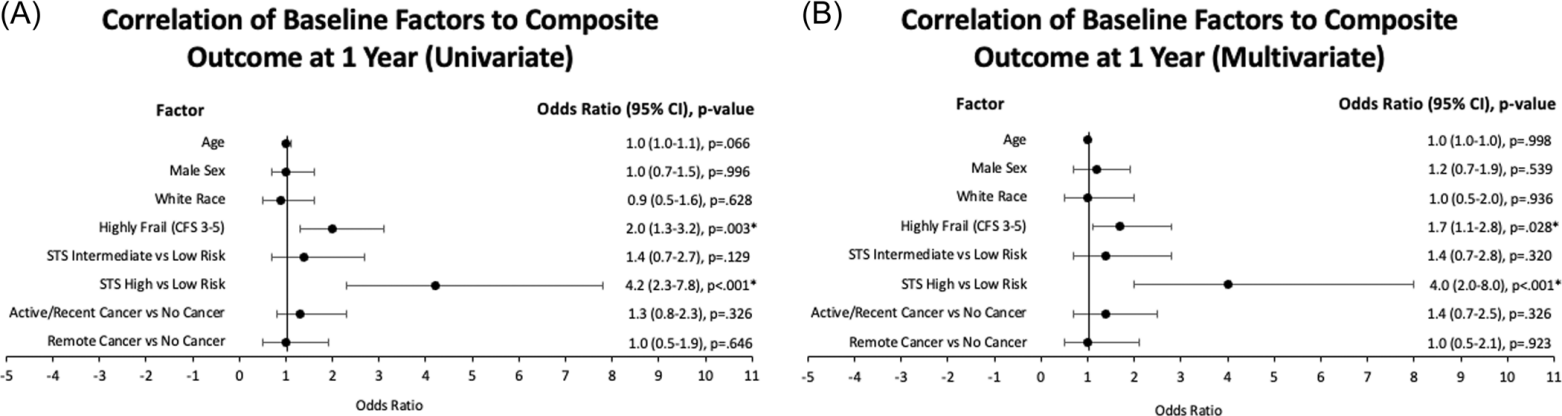
(A) Correlation of baseline factors to composite outcome at 1 year. With univariate analysis, the CFS (*p* = .003) and high‐risk STS scores (*p* < .000) were significantly associated with the composite outcome at 1 year for the overall TAVR population. (B) Multivariate model of baseline factors correlating with the composite outcome at 1 year. In the multivariate analysis, the CFS was 1.7 times more likely to be independently associated with the composite outcome (95% CI 1.1–2.8; *p* = .028), while high‐risk STS scores continued to be the strongest correlate with the composite outcome at 1 year (4.0 OR, 95% CI 2.0–8.0; *p* < .001). Neither active/recent cancer nor remote cancer history was significantly associated with the primary outcome. *Denotes statistically significant *p* values (*p* ≤ .05). CFS, composite frailty score; CI, confidence interval; OR, odds ratio; STS, Society of Thoracic Surgeons.

Figure 1 shows the univariate and multivariate regression analysis for the 1‐year composite outcome. These models included age, sex, race, STS score, frailty, and cancer history. With univariate analysis, high frailty (CFS 3–5) and high‐risk STS scores were significantly associated with a poor composite outcome at 1 year for the overall population (*p* = .003 and *p* < .00, respectively). Notably, active/recent cancer was not significantly associated with outcomes (1.3 OR, 95% CI: 0.8–2.3; *p* = .326) nor was remote cancer history (1.0 OR, 95% CI: 0.5–1.9; *p* = .646). In the multivariate regression, frailty continued to be independently associated with   outcomes, with 1.7 times the odds of meeting the primary composite outcome (95% CI: 1.1–2.8; *p* = .028). High‐risk STS scores had the strongest correlation with the composite outcome at 1 year (4.0 OR, 95% CI: 2.0–8.0; *p* < .001).

Given that frailty was significantly associated with the primary composite outcome in the overall population in both the univariate and multivariate analysis, further univariate analysis was conducted to see which components of the CFS were most impactful. The estimated odds ratio of the CFS as well as that of each individual frailty marker on 1‐year outcomes in each cohort are shown in Supporting Information: Tables 2a–d and the Central Illustration 1. Notably, a high CFS was not significantly related to any outcome in either the active/recent or remote cancer cohorts. Low hemoglobin significantly correlated with mortality in the active/recent cancer group, while poor cognition correlated with readmission rates in the active/recent and remote cancer groups. In the non‐cancer cohort, a high CFS correlated with a poor composite outcome (2.3 OR, 95% CI: 1.3–3.9; *p* = .004), mortality (2.0 OR, 95% CI: 1.0–3.8; *p* = .045), poor QOL (2.8 OR, 95% CI: 1.2–6.7; *p* = .018), and higher readmission rates (2.5 OR, 95% CI: 1.6–3.9; *p* < .001). Thirty‐day outcome data are depicted in Supporting Information: Tables 4, 5a–d, and 6 and Supporting Information: Figure 2.

**Central Illustration 1:**
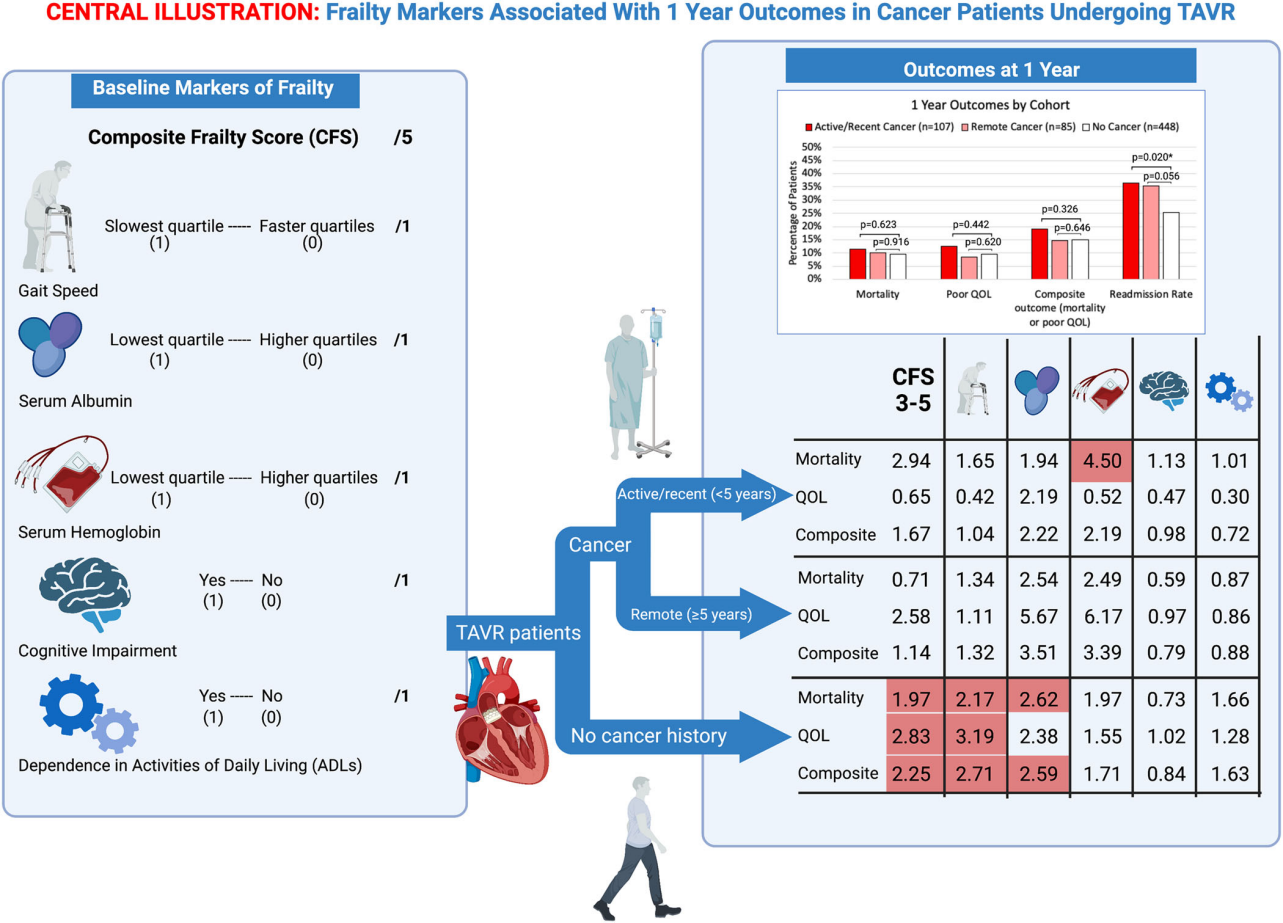
Frailty markers associated with 1‐year outcomes in cancer patients undergoing TAVR. Cancer status did not influence mortality, QOL, or a composite outcome of the two in this TAVR population. Overall, non‐cancer patients experienced significantly decreased readmission rates when compared to patients with a cancer history. Frailty was associated with poorer outcomes in the non‐cancer cohort, but its association was not significant in patients with a history of cancer. From left to right, the odds ratios were calculated by comparing CFS 3–5 to CFS 0–3, lowest quartile of gait speed to all other quartiles, lowest quartile of albumin to all other quartiles, lowest quartile of hemoglobin to all other quartiles, poor cognition to normal cognition, and functional dependence to functional independence. Red boxes denote statistically significant odds ratios. Created by biorender.com. QOL, quality of life; TAVR, transcatheter aortic valve replacement.

## 4 DISCUSSION

Multidisciplinary Heart Teams in TAVR programs may have heterogeneous patient populations with varying assessments of patient candidacy based on comorbidities and institutional experience. It is important to evaluate specific metrics of whether cancer history and frailty influence outcomes to discern those who would benefit most from TAVR. To our knowledge, our study is the first analysis of frailty metrics in patients with a history of cancer undergoing TAVR. In this single institution retrospective cohort study, we demonstrated that cancer history by itself does not affect frailty‐adjusted mortality and QOL outcomes at 1 year, while the CFS is significantly related to these outcomes in the overall population. However, when the cohort was stratified by cancer status, the CFS was not associated with outcomes in the active/recent cancer and remote cancer cohorts. Additionally, we observed that a history of cancer was associated with increased readmission rates at 1 year.

### 4.1 Impact of frailty on outcomes

In patients undergoing TAVR, frailty, as measured by various metrics, has been associated with increased mortality, increased disability, and decreased QOL.^13^, ^14^, ^15^ A multicenter, multinational study, the FRAILTY‐AVR study, compared seven frailty scales and found that the EFT, which includes chair rises, cognitive impairment, hemoglobin, and serum albumin, outperformed other frailty scales in predicting death and disability at 1 year.^14^ In a substudy of the PARTNER trial, which did not mention cancer prevalence, the authors found that the frail cohort had significantly increased mortality and a higher rate of the poor outcome (defined as death, KCCQ‐OS score <60, or a decrease of ≥10 points in the KCCQ‐OS score from baseline) 1 year after TAVR.^13^ Our study utilized a modified EFT, substituting gait speed for chair rises and incorporating functional dependence. Using a KCCQ‐OS score cutoff of 45 due to its correlation with NYHA functional class III/IV, we found similar rates of poor outcomes in our frail cohort. In accordance with the literature, our results show that patients in the higher frailty group had lower mean KCCQ‐OS scores and a greater percentage of KCCQ‐OS scores <45 at 30 days and 1 year.

In addition, our study found that a high CFS and high‐risk STS scores were associated with the composite outcome in the overall study population. This finding is in part explained by the positive correlation between STS scores and frailty at baseline since STS scores incorporate components of frailty. Further analysis illustrated that most individual frailty markers in this study failed to correlate with outcomes in either of our cancer cohorts. This may be due to selection bias, in which patients with a cancer history are more likely to be excluded due to perceived frailty and/or poor prognoses. Additionally, this could be due to the small sizes of our active/recent and remote cancer cohorts in comparison to our non‐cancer cohort. At our institution, most patients with a cancer history had chronic malignancies rather than fast‐growing cancers such as acute leukemia. Patients with chronic malignancies may not experience the same rate of declining ability to deal with stressors as patients with acute cancers and, therefore, may be similar to patients without a history of cancer in regards to TAVR related outcomes. Thus, it may be appropriate to presume that frailty has a similar effect on this patient population as the non‐cancer population and that effect may be seen in a larger cohort of patients.

Our study demonstrates that frailty should play a major factor in assessing candidacy in the general AS population for TAVR procedures; however, a history of cancer‐‐even the presence of active malignancy‐‐should not be an absolute contraindication. To ensure selection of appropriate candidates with a cancer history for TAVR, multidisciplinary input regarding overall cancer status and prognosis from oncology, cardio‐oncology, and geriatric specialists is imperative. TAVR should be considered for patients with an oncologic history after careful evaluation and optimization of their frailty as well as other comorbidities.

### 4.2 Limitations

Selection bias was present when considering our patient population and its retrospective nature. This was a single center study and as such, influenced by local procedural experience, selection bias, and referral pattern bias. Another limitation is that there is no frailty scale that is accepted as the gold standard. To address this, we used a modified version of the EFT, a frailty score which Afilalo et al.^14^ found to outperform other scores when identifying geriatric patients at high risk for poor TAVR outcomes. Additionally, we were limited by a subjective description of cognitive impairment and functional dependence when Katz scores or a MMSE/MOCA were not available in the EHR. Finally, there was significant variability on when the 30‐day and 1‐year follow‐up visits occurred for each patient. The mean number of days to the first follow‐up was 32.9 ± 11.7 and to the second follow‐up was 363.6 ± 51.0. While we had robust follow‐up data at 30 days, there were more missing values at 1 year especially regarding KCCQ data.

## 5 CONCLUSIONS

As the population of active and prior cancer patients continues to grow, there is increasing interest in understanding the impact of cancer and its treatments on cardiovascular disease. The prognostic value of cancer status, its associated frailty, and other comorbidities on outcomes following TAVR is a highly relevant topic which will likely represent a growing proportion of eligible patients for this intervention. Our study demonstrated that cancer status is not associated with mortality and QOL outcomes following TAVR, while increased frailty is strongly associated with worse outcomes in the overall TAVR population. We also found that frailty specifically within the cancer population was not associated with poorer outcomes, although selection bias and small cohort sizes may have played a role in this. Further investigation with greater numbers of active and prior cancer patients is warranted to elucidate whether frailty leads to poorer outcomes in patients with cancer undergoing TAVR.

## CONFLICT OF INTEREST

Eric H. Yang reports receiving research grants from CSL Behring, Boehringer Ingelheim and Eli Lilly, and consulting fees from Pfizer.

## Supporting information

Supporting information.

## Data Availability

The data that supports the findings of this study are available in the manuscript and supplementary material of this article.

## References

[1] Perrot N , Boekholdt SM , Mathieu P , Wareham NJ , Khaw KT , Arsenault BJ . Life's simple 7 and calcific aortic valve stenosis incidence in apparently healthy men and women. Int J Cardiol. 2018;269:226‐228. 10.1016/j.ijcard.2018.07.107 30054144 PMC6481556

[2] Lindman BR , Clavel MA , Mathieu P , et al. Calcific aortic stenosis. Nat Rev Dis Prim. 2016;2:16006. 10.1038/nrdp.2016.6 27188578 PMC5127286

[3] Faggiano P , Frattini S , Zilioli V , et al. Prevalence of comorbidities and associated cardiac diseases in patients with valve aortic stenosis. Potential implications for the decision‐making process. Int J Cardiol. 2012;159(2):94‐99. 10.1016/j.ijcard.2011.02.026 21376407

[4] Mangner N , Woitek FJ , Haussig S , et al. Impact of active cancer disease on the outcome of patients undergoing transcatheter aortic valve replacement. J Interv Cardiol. 2018;31(2):188‐196. 10.1111/joic.12458 29166702

[5] Popma JJ , Deeb GM , Yakubov SJ , et al. Transcatheter aortic‐valve replacement with a self‐expanding valve in low‐risk patients. N Engl J Med. 2019;380(18):1706‐1715. 10.1056/NEJMoa1816885 30883053

[6] Mack MJ , Leon MB , Thourani VH , et al. Transcatheter aortic‐valve replacement with a balloon‐expandable valve in low‐risk patients. N Engl J Med. 2019;380(18):1695‐1705. 10.1056/NEJMoa1814052 30883058

[7] Reardon MJ , Van Mieghem NM , Popma JJ , et al. Surgical or transcatheter aortic‐valve replacement in intermediate‐risk patients. N Engl J Med. 2017;376(14):1321‐1331. 10.1056/nejmoa1700456 28304219

[8] Smith CR , Leon MB , Mack MJ , et al. Transcatheter versus surgical aortic‐valve replacement in high‐risk patients. Surv Anesthesiol. 2012;56(1):4‐5. 10.1097/01.sa.0000410147.99581.d4 21639811

[9] Leon MB , Smith CR , Mack M , et al. Transcatheter aortic‐valve implantation for aortic stenosis in patients who cannot undergo surgery. N Engl J Med. 2010;363(17):1597‐1607. 10.1056/nejmoa1008232 20961243

[10] Leon MB , Smith CR , Mack MJ , et al. Transcatheter or surgical aortic‐valve replacement in intermediate‐risk patients. N Engl J Med. 2016;374(17):1609‐1620. 10.1056/nejmoa1514616 27040324

[11] Gill C , Lee M , Balanescu DV , et al. Transcatheter and surgical aortic valve replacement impact on outcomes and cancer treatment schedule. Int J Cardiol. 2021;326:62‐70. 10.1016/j.ijcard.2020.08.071 32858137

[12] Murphy AC , Koshy AN , Cameron W , et al. Transcatheter aortic valve replacement in patients with a history of cancer: periprocedural and long‐term outcomes. Catheter Cardiovasc Interv. 2020:1‐8. 10.1002/ccd.28969 32497385

[13] Green P , Arnold SV , Cohen DJ , et al. Relation of frailty to outcomes after transcatheter aortic valve replacement (from the PARTNER Trial). Am J Cardiol. 2015;116(2):264‐269. 10.1016/j.amjcard.2015.03.061 25963221 PMC4475494

[14] Afilalo J , Lauck S , Kim DH , et al. Frailty in older adults undergoing aortic valve replacement: the FRAILTY‐AVR Study. J Am Coll Cardiol. 2017;70(6):689‐700. 10.1016/j.jacc.2017.06.024 28693934

[15] Tzoumas A , Kokkinidis DG , Giannopoulos S , et al. Frailty in patients undergoing transcatheter aortic valve replacement: from risk scores to frailty‐based management. J Geriatr Cardiol. 2021;18(6):479‐486. 10.11909/j.issn.1671-5411.2021.06.002 34220976 PMC8220380

[16] Feldman D , Romashko M , Koethe B , et al. Comorbidity burden and adverse outcomes after transcatheter aortic valve replacement. J Am Coll Cardiol. 2020;75(11 Suppl 1):1423. 10.1016/S0735-1097(20)32050-7 PMC8200712 33960198

[17] Arnold SV , Spertus JA , Vemulapalli S , et al. Quality‐of‐life outcomes after transcatheter aortic valve replacement in an unselected population: a report from the STS/ACC transcatheter valve therapy registry. JAMA Cardiol. 2017;2(4):409‐416. 10.1001/jamacardio.2016.5302 28146260 PMC5408740

[18] Goldwater D , Altman NL . Frailty and heart failure ‐ American College of Cardiology. 2016. Accessed June 27, 2021. https://www.acc.org/latest-in-cardiology/articles/2016/08/05/08/40/frailty-and-heart-failure

[19] Ethun CG , Bilen MA , Jani AB , Maithel SK , Ogan K , Master VA . Frailty and cancer: implications for oncology surgery, medical oncology, and radiation oncology. CA Cancer J Clin. 2017;67(5):362‐377. 10.3322/caac.21406 28731537

[20] Harris PA , Taylor R , Minor BL , et al. The REDCap consortium: building an international community of software platform partners. J Biomed Inform. 2019;95:95. 10.1016/j.jbi.2019.103208 PMC7254481 31078660

[21] Harris PA , Taylor R , Thielke R , Payne J , Gonzalez N , Conde JG . Research electronic data capture (REDCap)—a metadata‐driven methodology and workflow process for providing translational research informatics support. J Biomed Inform. 2009;42(2):377‐381. 10.1016/j.jbi.2008.08.010 18929686 PMC2700030

[22] Skaar E , Eide LSP , Norekvål TM , et al. A novel geriatric assessment frailty score predicts 2‐year mortality after transcatheter aortic valve implantation. Eur Hear J Qual Care Clin Outcomes. 2019;5(2):153‐160. 10.1093/ehjqcco/qcy044 PMC6440438 30256921

[23] Green CP , Porter CB , Bresnahan DR , Spertus JA . Development and evaluation of the Kansas City cardiomyopathy questionnaire: a new health status measure for heart failure. J Am Coll Cardiol. 2000;35(5):1245‐1255. 10.1016/S0735-1097(00)00531-3 10758967

[24] Arnold SV , Spertus JA , Lei Y , et al. How to define a poor outcome after transcatheter aortic valve replacement: conceptual framework and empirical observations from the placement of aortic transcatheter valve (PARTNER) trial. Circ Cardiovasc Qual Outcomes. 2013;6(5):591‐597. 10.1161/CIRCOUTCOMES.113.000354 24021691 PMC4251553

[25] Spertus J , Peterson E , Conard MW , et al. Monitoring clinical changes in patients with heart failure: a comparison of methods. Am Heart J. 2005;150(4):707‐715. 10.1016/j.ahj.2004.12.010 16209970

[26] Spertus JA , Jones PG , Sandhu AT , Arnold SV . Interpreting the Kansas City Cardiomyopathy Questionnaire in Clinical Trials and Clinical Care: JACC State‐of‐the‐Art Review. J Am Coll Cardiol. 2020;76(20):2379‐2390. 10.1016/j.jacc.2020.09.542 33183512

[27] Arnold SV , Afilalo J , Spertus JA , et al. Prediction of poor outcome after transcatheter aortic valve replacement. J Am Coll Cardiol. 2016;68(17):1868‐1877. 10.1016/j.jacc.2016.07.762 27765189 PMC5119650

