# Supplementary material for: Impact of frailty on mortality and quality of life in patients with a history of cancer undergoing transcatheter aortic valve replacement
Source: Clin Cardiol. 2022 Oct 4;45(10):977–85. doi: 10.1002/clc.23927 (PMC9574730; doi:10.1002/clc.23927)
Supplement: Supplementary file 1 — Supporting information. [file CLC-45-977-s001.docx]

Supplemental Appendix

**Methods**

**Cancer Stratification**

A 5-year timepoint was chosen to align with oncology’s clinical practice to designate a patient as cancer free after 5 years of remission. Patients with nonmetastatic, nonmelanoma skin cancer were included in the non-cancer cohort. Patients with incidental findings of cancer from the pre-TAVR computed tomography (CT) evaluation were only included in the active/recent cancer cohort if their diagnoses were confirmed prior to TAVR.

**Frailty Assessment**

Cognitive impairment was defined as having a diagnosis of mild cognitive impairment, dementia (i.e., Alzheimer’s Disease), or an abnormal Mini-Mental State Examination (MMSE) or Montreal Cognitive Assessment (MoCA) score in the EHR at any time prior to the procedure date. A MMSE score ≤ 24 or MoCA score ≤ 25 were used as cutoffs. Functional dependence was defined as being dependent in any one of the 6 activities of daily living (ADLs) as noted in the EHR in the one year prior to the procedure date.

Serum albumin, hemoglobin, and 3 trials of the 5-meter walk test (5MWT) were collected as part of the pre-TAVR evaluation and were recorded in the TVT registry. 5MWT times were converted into gait speeds (m/s) and the average speed was used. Gait speed, serum albumin, and hemoglobin were divided into quartiles. Patients who were unable to walk were placed in the lowest quartile of gait speed. Cognitive impairment and functional dependence were binary variables.

For patients who were missing baseline gait speed, serum albumin, or hemoglobin, a CFS was created by summing the non-missing elements.

**Readmission Rates**

Only 1 readmission per patient was counted to derive the readmission rate. Elective admissions post-TAVR were excluded.

**Discussion**

*Impact of cancer history on outcomes.*

The increased focus on cancer history in TAVR registries reflects the growing interest of the relevant topic of cancer biology and/or treatments on TAVR-related outcomes. One such registry demonstrated that procedural mortality at 1 year was higher in the active cancer group compared to matched non-cancer controls with the increased mortality being attributed largely to noncardiac disease^1,2^. Additional studies have demonstrated that patients with active cancer had reduced 10-year survival rates, but similar 30-day survival rates to controls^2–4^. In contrast, a recent systematic review indicated that both short-term and long-term all-cause mortality were not significantly impacted by cancer history^5^. However, frailty metrics were not well defined in these studies.

Our data demonstrated that patients with remote cancer have a higher all-cause mortality at 30 days compared to the other two cohorts. The remote cancer patients were significantly older (82.7 ± 8.9 years) than the active/recent cancer patients (80.8 ± 9.7 years) and the non-cancer patients (79.7 ± 10.9 years). This result may also be explained by the sequelae of prior cancer treatments such as chemotherapy, which may have caused further comorbidity; however, survival and selection bias may also account for these findings. Additionally, with only 107 active/recent cancer patients and 85 remote cancer patients, our analysis was limited by study population size (i.e., reduced power). Our findings also show that neither an active nor remote cancer history was associated with a higher 1 year mortality, which may be attributed to the predominant inclusion of chronic malignancies, such as prostate and breast cancers. This is supported by our data, which revealed that the top 3 malignancies for our active/recent cancer cohort were multiple cancers (27%), prostate (21%), and breast (18%). The top 3 malignancies for our remote cancer cohort were breast (28%), prostate (17%), and colon (12%).

KCCQ scores have been used to evaluate QOL outcomes in TAVR studies. For instance, a report from the STS/ACC TVT registry using KCCQ scores, found that about 2 in 3 patients had a favorable health status outcome at 1 year post-TAVR^6^. However, there is limited data available on the use of KCCQ as an outcome measure in patients with a history of cancer undergoing TAVR. Our results show that cancer history does not influence QOL measured using KCCQ-OS scores at 30 days or 1 year.

In a study using data from the Nationwide Readmission Database, patients with cancer undergoing TAVR were found to have a significantly higher rate of readmission within 30 days post-discharge when compared to patients without cancer^7^. To our knowledge, there is no literature directly addressing the 1 year readmission rate. Our data differs from the current literature since our results show that there was no significant difference in readmission rates amongst the cohorts at 30 days. However, at 1 year, the non-cancer cohort had a significantly lower readmission rate compared to the other cohorts. The lack of difference in 30 day readmission rates may be attributed to patient selection. The 1 year results could also be attributed to patient selection where providers have a lower threshold to readmit patients with a cancer history who have undergone a recent cardiac procedure given their immunocompromised and hypercoagulable predisposition due to malignancy.

1. Mangner N, Woitek FJ, Haussig S, et al. Impact of active cancer disease on the outcome of patients undergoing transcatheter aortic valve replacement. *J Interv Cardiol*. 2018;31(2):188-196. doi:10.1111/joic.12458

2. Bendary A, Ramzy A, Bendary M, Salem M. Transcatheter aortic valve replacement in patients with severe aortic stenosis and active cancer: A systematic review and meta-analysis. *Open Hear*. 2020;7(1):1-10. doi:10.1136/openhrt-2019-001131

3. Landes U, Iakobishvili Z, Vronsky D, et al. Transcatheter Aortic Valve Replacement in Oncology Patients With Severe Aortic Stenosis. *JACC Cardiovasc Interv*. 2019;12(1):78-86. doi:10.1016/j.jcin.2018.10.026

4. Lind A, Totzeck M, Mahabadi AA, et al. Impact of Cancer in Patients Undergoing Transcatheter Aortic Valve Replacement: A Single-Center Study. *JACC CardioOncology*. 2020;2(5):735-743. doi:10.1016/j.jaccao.2020.11.008

5. Murphy AC, Koshy AN, Cameron W, et al. Transcatheter aortic valve replacement in patients with a history of cancer: Periprocedural and long-term outcomes. *Catheter Cardiovasc Interv*. 2020;(May):1-8. doi:10.1002/ccd.28969

6. Arnold S V., Spertus JA, Vemulapalli S, et al. Quality-of-life outcomes after transcatheter aortic valve replacement in an unselected population: A report from the STS/ACC transcatheter valve therapy registry. *JAMA Cardiol*. 2017;2(4):409-416. doi:10.1001/jamacardio.2016.5302

7. Jain V, Saad AM, Gad MM, et al. Outcomes of Cancer Patients Undergoing Transcatheter Aortic Valve Replacement. *JACC CardioOncology*. 2020;2(3):506-508. doi:10.1016/j.jaccao.2020.05.023

**Supplementary Table 1. Baseline Frailty Stratified by Cancer Status**

|  | **Active/Recent Cancer (n=107)** | **Remote Cancer (n=85)** | **No Cancer (n=448)** | **p-value** |
| --- | --- | --- | --- | --- |
| **Hemoglobin, g/dL (mean)** | 11.4 ± 2.2 | 12.0 ± 2.1 | 11.8 ± 2.2 | 0.145 |
| **Albumin, g/dL (mean)** | 3.9 ± 0.6 | 4.0 ± 0.5 | 4.0 ± 0.5 | 0.941 |
| **Gait speed (m/s)** |  |  |  |  |
| Trial 1 (mean) | 0.78 ± 0.27 | 0.71 ± 0.25 | 0.73 ± 0.31 | 0.386 |
| Trial 2 (mean) | 0.78 ± 0.29 | 0.71 ± 0.26 | 0.72 ± 0.30 | 0.207 |
| Trial 3 (mean) | 0.77 ± 0.26 | 0.7 ± 0.23 | 0.72 ± 0.30 | 0.403 |
| Average speed of 3 trials | 0.77 ± 0.27 | 0.7 ± 0.25 | 0.72 ± 0.30 | 0.288 |
| **Unable to walk** | 26 (24.3%) | 20 (23.5%) | 95 (21.2%) | 0.737 |
| **Dependent in any ADLs** | 26 (24.3%) | 22 (25.9%) | 100 (22.3%) | 0.737 |
| **Poor cognition** | 16 (15.0%) | 16 (18.8%) | 70 (15.6%) | 0.727 |
| **Composite frailty score (mean)** | 1.2 ± 0.9 | 1.3 ± 0.9 | 1.2 ± 1.0 | 0.445 |

ADL = activities of daily living

**Supplementary Table 2a. Correlation of Frailty Markers in Each Cohort to Composite Outcome at 1 Year**

|  | **Active/recent cancer**  **OR (95% CI) p-value** | **Cancer >5 years ago**  **OR (95% CI) p-value** | **No cancer**  **OR (95% CI) p-value** |
| --- | --- | --- | --- |
| **High CFS (3-5)** | 1.67 (0.62-4.48) p=0.312 | 1.14 (0.31-4.22) p=0.841 | 2.25 (1.30-3.87) p=0.004* |
| **Gait speed** | 1.04 (0.37-2.93) p=0.937 | 1.32 (0.38-4.54) p=0.658 | 2.71 (1.54-4.76) p=0.001* |
| **Albumin** | 2.22 (0.74-6.67) p=0.157 | 3.51 (0.94-13.10) p=0.062 | 2.59 (1.46-4.62) p=0.001* |
| **Hemoglobin** | 2.19 (0.81-5.91) p=0.121 | 3.39 (0.92-12.52) p=0.067 | 1.71 (0.96-3.07) p=0.071 |
| **Poor cognition** | 0.98 (0.25-3.82) p=0.974 | 0.79 (0.15-4.00) p=0.772 | 0.84 (0.4-1.80) p=0.662 |
| **Dependence in ADLs** | 0.72 (0.22-2.37) p=0.585 | 0.88 (0.21-3.59) p=0.855 | 1.63 (0.91-2.92) p=0.100 |

ADL = activities of daily living; CFS = composite frailty score

**Supplementary Table 2b. Correlation of Frailty Markers in Each Cancer Cohort to Mortality at 1 Year**

|  | **Active/recent cancer**  **OR (95% CI) p-value** | **Cancer >5 years ago**  **OR (95% CI) p-value** | **No cancer**  **OR (95% CI) p-value** |
| --- | --- | --- | --- |
| **High CFS (3-5)** | 2.94 (0.86-10.03) p=0.085 | 0.71 (0.13-3.79) p=0.689 | 1.97 (1.02-3.81) p=0.045* |
| **Gait speed** | 1.65 (0.46-5.83) p=0.440 | 1.34 (0.31-5.83) p=0.692 | 2.17 (1.08-4.35) p=0.029* |
| **Albumin** | 1.94 (0.50-7.54) p=0.341 | 2.54 (0.54-12.01) p=0.240 | 2.62 (1.32-5.20) p=0.006* |
| **Hemoglobin** | 4.50 (1.25-16.19) p=0.021* | 2.49 (0.53-11.66) p=0.248 | 1.97 (0.99-3.92) p=0.055 |
| **Poor cognition** | 1.13 (0.22-5.71) p=0.884 | 0.59 (0.07-5.21) p=0.637 | 0.73 (0.27-1.93) p=0.522 |
| **Dependence in ADLs** | 1.01 (0.25-4.07) p=0.984 | 0.87 (0.16-4.66) p=0.868 | 1.66 (0.82-3.34) p=0.157 |

ADL = activities of daily living; CFS = composite frailty score

**Supplementary Table 2c. Correlation of Frailty Markers in Each Cancer Cohort to QOL at 1 Year**

|  | **Active/recent cancer**  **OR (95% CI) p-value** | **Cancer >5 years ago**  **OR (95% CI) p-value** | **No cancer**  **OR (95% CI) p-value** |
| --- | --- | --- | --- |
| **High CFS (3-5)** | 0.65 (0.12-3.53) p=0.617 | 2.58 (0.33-20.48) p=0.369 | 2.83 (1.20-6.70) p=0.018* |
| **Gait speed** | 0.42 (0.07-2.36) p=0.322 | 1.11 (0.14-8.64) p=0.924 | 3.19 (1.30-7.80) p=0.011* |
| **Albumin** | 2.19 (0.43-11.12) p=0.344 | 5.67 (0.66-48.33) p=0.113 | 2.38 (0.94-6.06) p=0.068 |
| **Hemoglobin** | 0.52 (0.10-2.84) p=0.454 | 6.17 (0.72-52.49) p=0.096 | 1.55 (0.58-4.15) p=0.383 |
| **Poor cognition** | 0.47 (0.05-4.20) p=0.501 | 0.97 (0.09-10.32) p=0.980 | 1.02 (0.33-3.16) p=0.970 |
| **Dependence in ADLs** | 0.30 (0.03-2.64) p=0.279 | 0.86 (0.08-9.11) p=0.901 | 1.28 (0.50-3.25) p=0.603 |

ADL = activities of daily living; CFS = composite frailty score; QOL= quality of life

**Supplementary Table 2d. Correlation of Frailty Markers in Each Cancer Cohort to Readmission Rates at 1 Year**

|  | **Active/recent cancer**  **OR (95% CI) p-value** | **Cancer >5 years ago**  **OR (95% CI) p-value** | **No cancer**  **OR (95% CI) p-value** |
| --- | --- | --- | --- |
| **High CFS (3-5)** | 1.73 (0.77-3.91) p=0.188 | 1.22 (0.47-3.17) p=0.685 | 2.48 (1.58-3.89) p<0.000* |
| **Gait speed** | 1.36 (0.59-3.14) p=0.471 | 1.13 (0.45-2.84) p=0.794 | 2.98 (1.89-4.68) p<0.000* |
| **Albumin** | 1.72 (0.69-4.29) p=0.245 | 0.97 (0.32-2.97) p=0.961 | 2.03 (1.25-3.28) p=0.004* |
| **Hemoglobin** | 2.05 (0.89-4.70) p=0.092 | 1.64 (0.57-4.72) p=0.363 | 2.02 (1.25-3.26) p=0.004* |
| **Poor cognition** | 7.11 (2.10-24.03) p=0.002* | 4.08 (1.31-12.74) p=0.015* | 1.23 (0.69-2.17) p=0.483 |
| **Dependence in ADLs** | 1.39 (0.56-3.42) p=0.476 | 2.32 (0.86-6.26) p=0.098 | 1.65 (1.02-2.68) p=0.043* |

ADL = activities of daily living; CFS = composite frailty score

**Supplementary Table 3. Non-Valve and Valve Related Readmissions at 1 Year**

|  | **Active/Recent Cancer** | **Remote Cancer** | **No Cancer** |
| --- | --- | --- | --- |
| **Non-Valve Related Readmissions** | 55 (98.2%) | 42 (97.7%) | 162 (92.6%) |
| **Valve Related Readmissions** | 1 (1.8%) | 1 (2.3%) | 13 (7.4%) |

Further analysis with a chi-square test was not performed given low observed values of valve related readmissions.

**Supplementary Table 4. 30 Day Outcomes Stratified by Cancer Status**

|  | **Total Population (n=640)** | **Active/Recent Cancer (n=107)** | **Remote Cancer (n=85)** | **No Cancer (n=448)** | **p-value** |
| --- | --- | --- | --- | --- | --- |
| **30 Day Mortality** | 10 (1.6%) | 2 (1.9%) | 4 (4.7%) | 4 (0.9%) | 0.032* |
| **30 Day KCCQ-OS (mean)** | 78.98 (22.2%) | 77.52 (20.9%) | 80.53 (21.1%) | 79.05 (22.8%) | 0.669 |
| **Patients with KCCQ-OS<45 at 30 Days** | 60 (10.4%) | 8 (8.1%) | 7 (9.2%) | 45 (11.2%) | 0.624 |
| **Patients with KCCQ-OS Drop >10 at 30 days** | 36 (6.5%) | 6 (6.5%) | 5 (6.7%) | 25 (6.4%) | 0.997 |
| **Readmission Rates at 30 Days** | 93 (14.5%) | 20 (18.7%) | 9 (10.6%) | 64 (14.3%) | 0.276 |
| **Patients with Poor Quality of Life at 30 Days** | 73 (12.6%) | 13 (13.1%) | 10 (13.4%) | 50 (12.4%) | 0.970 |
| **Patients with Poor Composite Outcomes at 30 Days** | 83 (13.0%) | 15 (14.0%) | 14 (16.5%) | 54 (12.1%) | 0.507 |

KCCQ-OS = Kansas City Cardiomyopathy Questionnaire overall summary

**Supplementary Table 5a. Correlation of Frailty Markers in Each Cohort to Composite Outcome at 30 Days**

|  | **Active/recent cancer**  **OR (95% CI) p-value** | **Cancer >5 years ago**  **OR (95% CI) p-value** | **No cancer**  **OR (95% CI) p-value** |
| --- | --- | --- | --- |
| **High CFS (3-5)** | 1.72 (0.57-5.19) p=0.334 | 3.93 (1.20-12.85) p=0.024* | 1.87 (1.04-3.36) p=0.037* |
| **Gait speed** | 1.34 (0.43-4.18) p=0.611 | 2.67 (0.81-8.83) p=0.108 | 1.59 (0.89-2.84) p=0.121 |
| **Albumin** | 1.38 (0.42-4.51) p=0.593 | 1.88 (0.50-7.07) p=0.350 | 1.37 (0.72-2.62) p=0.336 |
| **Hemoglobin** | 2.53 (0.84-7.67) p=0.101 | 1.02 (0.25-4.12) p=0.980 | 1.85 (1.00-3.41) p=0.051 |
| **Poor cognition** | 0.86 (0.17-4.22) p=0.850 | 4.58 (1.31-16.00) p=0.017* | 1.27 (0.60-2.65) p=0.533 |
| **Dependence in ADLs** | 0.44 (0.09-2.07) p=0.297 | 1.18 (0.33-4.22) p=0.802 | 1.25 (0.65-2.41) p=0.498 |

ADL = activities of daily living; CFS = composite frailty score

**Supplementary Table 5b. Correlation of Frailty Markers in Each Cancer Cohort to Mortality at 30 Days**

|  | **Active/recent cancer**  **OR (95% CI) p-value** | **Cancer >5 years ago**  **OR (95% CI) p-value** | **No cancer**  **OR (95% CI) p-value** |
| --- | --- | --- | --- |
| **High CFS (3-5)** | 1.84 (0.11-30.24) p=0.670 | >999 (<0.001->999) p=0.957 | 7.66 (0.79-74.30) p=0.079 |
| **Gait speed** | 1.29 (0.08-21.29) p=0.858 | >999 (<0.001->999) p=0.943 | 4.21 (0.43-40.79) p=0.215 |
| **Albumin** | 2.68 (0.16-44.50) p=0.492 | 4.13 (0.54-31.77) p=0.176 | <0.001 (<0.001->999) p=0.964 |
| **Hemoglobin** | <0.001 (<0.001->999) p=0.954 | 1.26 (0.12-12.84) p=0.848 | 1.13 (0.12-11.00) p=0.915 |
| **Poor cognition** | <0.001 (<0.001->999) p=0.970 | 1.47 (0.14-15.10) p=0.748 | <0.001 (<0.001->999) p=0.971 |
| **Dependence in ADLs** | <0.001 (<0.001->999) p=0.962 | 3.05 (0.40-23.08) p=0.280 | 1.16 (0.12-11.29) p=0.897 |

ADL = activities of daily living; CFS = composite frailty score

**Supplementary Table 5c. Correlation of Frailty Markers in Each Cancer Cohort to QOL at 30 Days**

|  | **Active/recent cancer**  **OR (95% CI) p-value** | **Cancer >5 years ago**  **OR (95% CI) p-value** | **No cancer**  **OR (95% CI) p-value** |
| --- | --- | --- | --- |
| **High CFS (3-5)** | 1.68 (0.52-5.47) p=0.386 | 2.27 (0.56-9.10) p=0.249 | 1.76 (0.95-3.28) p=0.072 |
| **Gait speed** | 1.33 (0.39-4.51) p=0.644 | 1.70 (0.44-6.49) p=0.441 | 1.51 (0.82-2.78) p=0.183 |
| **Albumin** | 1.20 (0.33-4.34) p=0.781 | 1.35 (0.25-7.40) p=0.729 | 1.59 (0.82-3.09) p=0.170 |
| **Hemoglobin** | 3.57 (1.07-11.96) p=0.039* | 1.02 (0.19-5.38) p=0.982 | 1.98 (1.04-3.74) p=0.037* |
| **Poor cognition** | 1.02 (0.20-5.15) p=0.980 | 7.25 (1.71-30.70) p=0.007* | 1.45 (0.68-3.07) p=0.336 |
| **Dependence in ADLs** | 0.50 (0.10-2.42) p=0.387 | 0.85 (0.16-4.44) p=0.847 | 1.28 (0.65-2.53) p=0.477 |

ADL = activities of daily living; CFS = composite frailty score; QOL= quality of life

**Supplementary Table 5d. Correlation of Frailty Markers in Each Cancer Cohort to Readmission Rates at 30 Days**

|  | **Active/recent cancer**  **OR (95% CI) p-value** | **Cancer >5 years ago**  **OR (95% CI) p-value** | **No cancer**  **OR (95% CI) p-value** |
| --- | --- | --- | --- |
| **High CFS (3-5)** | 0.97 (0.35-2.69) p=0.958 | 1.15 (0.26-5.01) p=0.850 | 3.00 (1.75-5.16) p<0.000* |
| **Gait speed** | 0.78 (0.27-2.23) p=0.645 | 1.65 (0.41-6.67) p=0.480 | 3.34 (1.89-5.89) p<0.000* |
| **Albumin** | 0.84 (0.27-2.61) p=0.765 | 2.07 (0.46-9.32) p=0.343 | 1.64 (0.91-2.95) p=0.098 |
| **Hemoglobin** | 1.04 (0.37-2.89) p=0.940 | 3.54 (0.84-14.91) p=0.084 | 2.17 (1.23-3.82) p=0.008* |
| **Poor cognition** | 3.30 (1.03-10.54) p=0.044* | 2.42 (0.54-10.96) p=0.250 | 1.14 (0.56-2.32) p=0.710 |
| **Dependence in ADLs** | 1.44 (0.49-4.22) p=0.511 | 2.58 (0.62-10.63) p=0.190 | 1.89 (1.06-3.36) p=0.031* |

ADL = activities of daily living; CFS = composite frailty score

**Supplementary Table 6. 30 Day and 1 Year Outcomes Stratified by Frailty**

|  | **Lower Frailty (CFS of 0-2) (n=448)** | **Higher Frailty (CFS of 3-5) (n=192)** | **p-value** |
| --- | --- | --- | --- |
| **Mortality** |  |  |  |
| 30 days | 2 (0.5%) | 8 (4.2%) | 0.001* |
| 1 year | 35 (8.0%) | 26 (14.2%) | 0.019* |
| **KCCQ-OS scores, mean** |  |  |  |
| 30 days | 81.8 ± 20.7 | 71.9 ± 24.2 | <0.001* |
| 1 year | 84.5 ± 19.4 | 78.8 ± 23.5 | 0.018* |
| **Patients with KCCQ-OS<45** |  |  |  |
| 30 days | 34 (8.2%) | 26 (15.9%) | 0.007* |
| 1 year | 15 (5.7%) | 11 (11.2%) | 0.071 |
| **Poor composite outcome** |  |  |  |
| 30 days | 46 (10.3%) | 37 (19.3%) | 0.002* |
| 1 year | 56 (12.8%) | 41 (22.4%) | 0.003* |

CFS = composite frailty score; KCCQ-OS = Kansas City Cardiomyopathy Questionnaire overall summary

**Supplementary Figure 1: Flow Chart of Patient Selection Process**

A total of 640 patients were eligible for analysis. The most common reason for ineligibility was not undergoing a transcatheter aortic valve replacement. These patients were included in the TVT registry due to mitral valve procedures. Of note, only 477 (74.5%) patients had both 30 days and 1 year follow up data.

TVT = transcatheter valve therapy

**
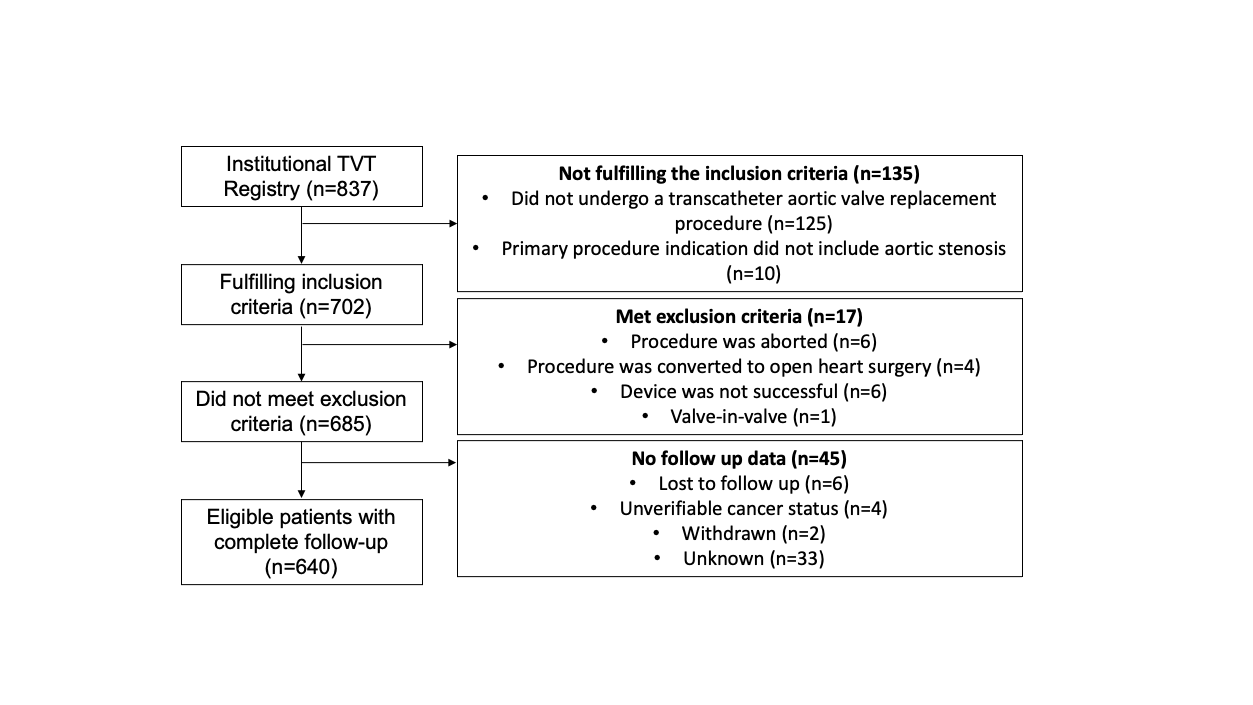
**

**Supplementary Figure 2a: Correlation of Baseline Factors to Composite Outcome at 30 Days**

With univariate analysis, only the CFS (p=0.002) was significantly correlated with the composite outcome at 30 days for the overall population.

CFS = composite frailty score; STS = Society of Thoracic Surgeons

**Supplementary Figure 2b: Multivariate Model of Baseline Factors correlating with the Composite Outcome at 30 Days**

In the multivariate analysis, the CFS continued to be independently associated with outcomes with 2.0 times the odds of meeting criteria for the primary composite outcome (95% CI 1.2 to 3.3; p=0.007). Active/recent cancer history was not significantly associated with outcomes (1.0 OR, 95% CI 0.5 to 1.9; p=0.969) nor was remote cancer history (1.4 OR, 95% CI 0.7 to 2.7; p=0.378).

CFS = composite frailty score; STS = Society of Thoracic Surgeons
